# The SARS-Unique Domain (SUD) of SARS Coronavirus Contains Two Macrodomains That Bind G-Quadruplexes

**DOI:** 10.1371/journal.ppat.1000428

**Published:** 2009-05-15

**Authors:** Jinzhi Tan, Clemens Vonrhein, Oliver S. Smart, Gerard Bricogne, Michela Bollati, Yuri Kusov, Guido Hansen, Jeroen R. Mesters, Christian L. Schmidt, Rolf Hilgenfeld

**Affiliations:** 1 Institute of Biochemistry, Center for Structural and Cell Biology in Medicine, University of Lübeck, Lübeck, Germany; 2 Global Phasing Ltd., Sheraton House, Castle Park, Cambridge, United Kingdom; 3 Laboratory for Structural Biology of Infection and Inflammation, c/o DESY, Hamburg, Germany; Institut Pasteur, France

## Abstract

Since the outbreak of severe acute respiratory syndrome (SARS) in 2003, the three-dimensional structures of several of the replicase/transcriptase components of SARS coronavirus (SARS-CoV), the non-structural proteins (Nsps), have been determined. However, within the large Nsp3 (1922 amino-acid residues), the structure and function of the so-called SARS-unique domain (SUD) have remained elusive. SUD occurs only in SARS-CoV and the highly related viruses found in certain bats, but is absent from all other coronaviruses. Therefore, it has been speculated that it may be involved in the extreme pathogenicity of SARS-CoV, compared to other coronaviruses, most of which cause only mild infections in humans. In order to help elucidate the function of the SUD, we have determined crystal structures of fragment 389–652 (“SUD_core_”) of Nsp3, which comprises 264 of the 338 residues of the domain. Both the monoclinic and triclinic crystal forms (2.2 and 2.8 Å resolution, respectively) revealed that SUD_core_ forms a homodimer. Each monomer consists of two subdomains, SUD-N and SUD-M, with a macrodomain fold similar to the SARS-CoV X-domain. However, in contrast to the latter, SUD fails to bind ADP-ribose, as determined by zone-interference gel electrophoresis. Instead, the entire SUD_core_ as well as its individual subdomains interact with oligonucleotides known to form G-quadruplexes. This includes oligodeoxy- as well as oligoribonucleotides. Mutations of selected lysine residues on the surface of the SUD-N subdomain lead to reduction of G-quadruplex binding, whereas mutations in the SUD-M subdomain abolish it. As there is no evidence for Nsp3 entering the nucleus of the host cell, the SARS-CoV genomic RNA or host-cell mRNA containing long G-stretches may be targets of SUD. The SARS-CoV genome is devoid of G-stretches longer than 5–6 nucleotides, but more extended G-stretches are found in the 3′-nontranslated regions of mRNAs coding for certain host-cell proteins involved in apoptosis or signal transduction, and have been shown to bind to SUD *in vitro*. Therefore, SUD may be involved in controlling the host cell's response to the viral infection. Possible interference with poly(ADP-ribose) polymerase-like domains is also discussed.

## Author Summary

The genome of the SARS coronavirus codes for 16 non-structural proteins that are involved in replicating this huge RNA (approximately 29 kilobases). The roles of many of these in replication (and/or transcription) are unknown. We attempt to derive conclusions concerning the possible functions of these proteins from their three-dimensional structures, which we determine by X-ray crystallography. Non-structural protein 3 contains at least seven different functional modules within its 1922-amino-acid polypeptide chain. One of these is the so-called SARS-unique domain, a stretch of about 338 residues that is completely absent from any other coronavirus. It may thus be responsible for the extraordinarily high pathogenicity of the SARS coronavirus, compared to other viruses of this family. We describe here the three-dimensional structure of the SARS-unique domain and show that it consists of two modules with a known fold, the so-called macrodomain. Furthermore, we demonstrate that these domains bind unusual nucleic-acid structures formed by consecutive guanosine nucleotides, where four strands of nucleic acid are forming a superhelix (so-called G-quadruplexes). SUD may be involved in binding to viral or host-cell RNA bearing this peculiar structure and thereby regulate viral replication or fight the immune response of the infected host cell.

## Introduction

**Figure 1:**
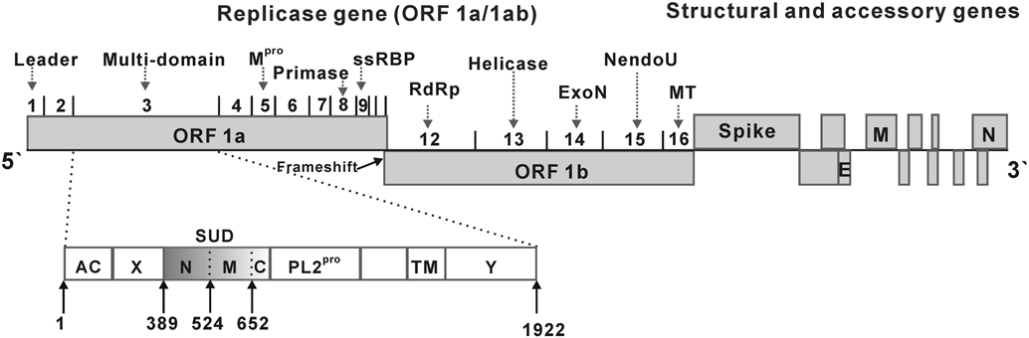
Genome organisation of SARS-CoV. Nsp3 and full-length SUD with subdomains N, M, and C are highlighted. M^pro^, main (or 3CL) protease; ssRBP, single-stranded RNA-binding protein; RdRp, RNA-dependent RNA polymerase; ExoN, exonuclease; NendoU, uridine-specific endoribonuclease; MT, methyltransferase; Spike, spike protein; E, envelope protein; M, membrane (matrix) protein; N, nucleocapsid protein; Ac, acidic domain; X, X-domain; SUD, SARS-unique domain; PL2^pro^, papain-like protease; TM, transmembrane region; Y, Y-domain.

The SARS coronavirus (SARS-CoV) is much more pathogenic for humans than any other coronavirus. Therefore, protein domains encoded by the SARS-CoV genome that are absent in other coronaviruses are of particular interest, because they may be responsible for the extraordinary virulence. The most prominent such domain has been identified by bioinformatics as part of non-structural protein 3 (Nsp3) of the virus and appropriately named the “SARS-unique domain” (SUD) [1]. With a molecular mass of 213 kDa, Nsp3 is the largest of the non-structural proteins of SARS coronavirus (see Figure 1). Comprising 1922 amino-acid residues (polyprotein 1a/1ab residues Ala819 to Gly2740), SARS-CoV Nsp3 is larger than the entire replicase of *Picornaviridae*. It contains at least seven subdomains [2]: An N-terminal acidic domain (Ac, also called Nsp3a); an X-domain (also designated as ADRP, or Nsp3b); the SUD (Nsp3c); a papain-like proteinase, PL2^pro^ (also called Nsp3d); and additional domains (Nsp3e–g) that include a transmembrane (TM) region.

At present, it is completely unclear whether and how the individual domains of Nsp3 interact with one another or with other components of the coronaviral replicase complex. Also, some of them possibly recognize proteins of the infected host cell [2]. In the absence of functional data on these domains, attempts have been made to derive their possible biological role from their three-dimensional structures (see [3] for a review). The NMR structure of an N-terminal fragment of the acidic domain (Nsp3a) has revealed a ubiquitin-like fold complemented by two additional short α-helices ([4], PDB code 2IDY). NMR chemical-shift analysis suggested that these non-canonical structural elements might bind single-stranded RNA with some specificity for AUA-containing sequences, although the K_D_ values observed are relatively high (∼20 µM). Interestingly, a second ubiquitin-like domain occurs in Nsp3, as part of the papain-like proteinase (PL2^pro^, Nsp3d, [5]; PDB code 2FE8). The PL2^pro^ cleaves the viral polyprotein after two consecutive glycine residues to release Nsp1, Nsp2, and Nsp3, respectively (The remaining cleavage reactions are performed by the coronaviral main proteinase (M^pro^; [6]–[8])). In addition to its proteolytic activities on the N-terminal third of the polyproteins, the SARS-CoV PL2^pro^ has also been shown to be a deubiquitinating enzyme [9]–[12]. Lindner *et al*. [13] have shown that in addition to its proteolytic and deubiquitinating activity, the SARS-CoV PL2^pro^ acts as a de-ISGylating enzyme. Induction of ISG15 and its subsequent conjugation to proteins protects cells from the effects of viral infection [14],[15]. Since the ISG15 gene is induced by interferon as part of the antiviral response of the innate immune system, the de-ISGylation activity of Nsp3d could explain the suppression of the interferon response by the papain-like protease, in addition to a possible direct interaction between the PL2^pro^ and IRF3 [16].

Among the subdomains of the Nsp3 multidomain protein, there is also the so-called “X- domain” (Nsp3b), which shows structural homology to macrodomains. The latter name refers to the non-histone-like domain of the histone macro2A [17]–[19]. In animal cells, such domains are occasionally physically associated with enzymes involved in ADP-ribosylation or ADP-ribose metabolism. Because of this linkage and on the basis of sequence similarity to Poa1p, a yeast protein involved in the removal of the 1″-phosphate group from ADP-ribose 1″-phosphate (a late step in tRNA splicing; [20]), it has been proposed that the coronaviral X-domains may have the function of ADP-ribose-1″-phosphatases (ADRPs; [21]). The crystal structures of X-domains of SARS-CoV [22],[23] as well as of HCoV 229E and Infectious Bronchitis Virus (IBV) [24] show that the protein has the three-layer α/β/α fold characteristic of the macrodomains.

Embedded between the X-domain (Nsp3b) and the PL2^pro^ (Nsp3d), the SARS-unique domain (SUD; Nsp3c) fails to show sequence relationship to any other protein in the databases [1]. We have produced full-length SUD (residues 389 to 726 of Nsp3), and a more stable, shortened 264-residue version (residues 389 to 652; henceforth called SUD_core_), by expression in *Escherichia coli*. This definition of the boundaries of the SUD is based on the structural results described here. We report crystallization of SUD_core_ and its X-ray structure in two crystal forms, at 2.2 and 2.8 Å resolution, respectively. The structure turns out to consist of two further copies of the macrodomain, in spite of the complete absence of sequence similarity. In addition, we demonstrate that each of the subdomains binds G-quadruplexes, both in DNA and RNA fragments, and that selected mutations of lysine residues in the first subdomain, SUD-N, lead to reduced nucleic-acid binding, whereas those in the second subdomain, SUD-M, abolish it.

## Results

### Quality of the structural models

Out of the many SUD constructs designed and tested by us, SUD_core_ (Nsp3 residues 389–652) turned out to be relatively stable and could be crystallized (Table 1). Two crystal forms were observed under identical crystallization conditions: Form-1 crystals were monoclinic (space group P2_1_, two SUD_core_ molecules per asymmetric unit) and diffracted X-rays to 2.2 Å resolution; form-2 crystals were triclinic (space group P1, four SUD_core_ molecules per asymmetric unit) and diffracted to 2.8 Å. Both structures were determined by molecular replacement (see Materials and Methods). The r.m.s. deviations (on Cα atoms) between the models derived from the two different crystal structures are around 0.7 Å.

**Table 1:** Data collection and refinement statistics.

	Monoclinic crystal form	Triclinic crystal form
**Data collection**		
Wavelength (Å)	1.25485	1.04123
Resolution (Å)	28.25–2.22 (2.34–2.22)	33.33–2.80 (2.96–2.80)
Space group	P2_1_	P1
Unit-cell parameters		
a (Å)	46.36	68.68
b (Å)	68.55	75.52
c (Å)	94.21	80.54
α (°)	90.00	77.16
β (°)	99.17	75.61
γ (°)	90.00	74.48
Solvent content (%, v/v)	51	63
Overall reflections	166585 (7062)	101963 (9416)
Unique reflections	26598 (2508)	34003 (4086)
Multiplicity	6.3 (2.8)	3.0 (2.3)
Completeness (%)	92.1 (61.0)	93.1 (76.6)
*R*_merge_ a	0.055 (0.373)	0.075 (0.252)
I/σ(I)	16.9 (2.0)	8.8 (2.0)
*R*_pim_ b	0.025 (0.316)	0.056 (0.252)
**Refinement**		
*R*_cryst_ c	0.211	0.223
*R*_free_ c	0.268	0.240
r.m.s.d. from ideal geometry		
bonds (Å)	0.009	0.008
angles (°)	1.295	1.188
Ramachandran plot regions		
Most favored (%)	94.8	94.7
Additionally allowed (%)	4.6	4.6
Outlier (%)	0.6	0.7

Values in parentheses are for the highest resolution shell.

a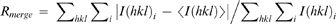, where *I*(*hkl*) is the intensity of reflection *hkl* and 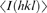 is the average intensity over all equivalent reflections.

b *R*_pim_ is the precision-indicating merging *R* factor [78].

c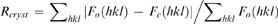. *R_free_* was calculated for a test set of reflections (5%) omitted from the refinement.

The models have good stereochemistry (Table 1). 94.7% of the amino-acid residues are in the favoured regions of the Ramachandran plot and 4.6% are in allowed regions. 0.6% are outliers. In all six independent copies of the SUD_core_ monomer, residue Val611 adopts forbidden conformational angles. This residue is located in a turn described by the polypeptide chain where it leaves the subdomain interface (see below) and reaches the surface of the molecule. The side chain makes a hydrophobic contact across the subdomain interface and is also contacting the side chain of Phe406 of a symmetry-related SUD_core_ dimer in the crystal lattice in the monoclinic crystal form (this also applies to two of the four monomers in the triclinic form).

### Overall structure

**Figure 2:**
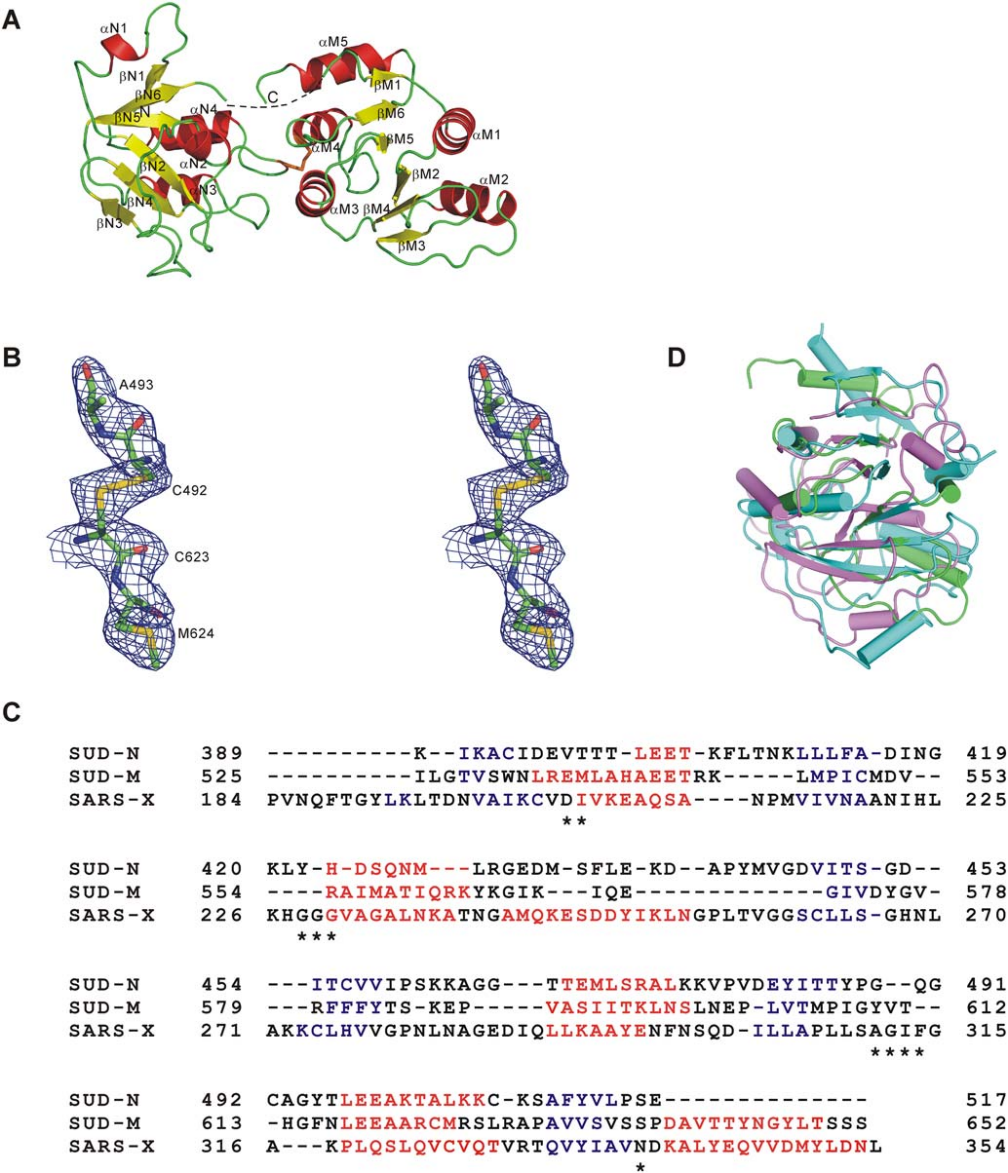
Structure of the SUD_core_ monomer and comparison with the SARS-CoV X-domain. (A) Ribbon representation of the SUD_core_ structure (residues 389–652 of Nsp3). The flexible linker connecting the two macrodomains is indicated by a dotted line. The disulfide bond between cysteines 492 of SUD-N and 623 of SUD-M is shown in orange. (B) Stereo image of the 2F_o_–F_c_ electron-density map (1σ above the mean) around the disulfide bond connecting the SUD-N and SUD-M subdomains. (C) Structure-based sequence alignment of the SUD_core_ subdomains N (SUD-N) and M (SUD-M), and the SARS-CoV X-domain (SARS-X). α-Helices and β-strands are marked red and blue, respectively. Residues 518–524 form the linker between the two SUD subdomains and have not been included in the alignment. Asterisks mark SARS-X residues involved in binding of ADP-ribose. (D) Superimposition of the structures of the SUD-N (violet) and SUD-M (green) subdomains with the SARS-CoV X-domain (cyan).

SUD_core_ exhibits a two-domain architecture (Figure 2A). The N-terminal subdomain (SUD-N) comprises Nsp3 residues 389–517, and the C-terminal subdomain of SUD_core_ contains residues 525–652. We call the latter the “middle SUD subdomain”, or SUD-M, because full-length SUD has a C-terminal extension of 74 residues compared to SUD_core_. The SUD-N and SUD-M subdomains have a similar fold and can be superimposed with an r.m.s.d. of 3.3–3.4 Å (based on Cα positions); they share 11% sequence identity (see Figure 2C for a structural alignment). Of the 14 amino-acid residues identical between the two subdomains, four form a conserved Leu-Glu-Glu-Ala motif at the N-terminus of helix α4. The linker between the two subdomains (residues 518–524) has no visible electron density. This is due to elevated mobility of the linker, rather than proteolytic cleavage, since we showed by SDS-PAGE of dissolved crystals that the SUD_core_ polypeptide (in the presence of β-mercaptoethanol) has the apparent molecular mass to be expected (∼29 kDa; not shown). In addition to the linker, SUD-N and SUD-M are connected by a disulfide bond between cysteines 492 and 623 (Figure 2B). Disulfide bonds are rare in cytosolic proteins, but in coronaviral Nsps, examples of such bonds have been reported [25],[26].

The fold of each SUD subdomain is that of a macrodomain (Figure 2A). Macrodomains consist of a largely parallel central β-sheet surrounded by 4–6 α-helices. The order of regular secondary-structure elements in SUD-N is βN1-αN1-βN2-αN2-βN3-βN4-αN3-βN5-αN4-βN6, and in SUD-M βM1-αM1-βM2-αM2-βM3-βM4-αM3-βM5-αM4-βM6-αM5. The topology of the β-strands is β1–β6–β5–β2–β4–β3, all of which are parallel except β3 (Figure 2A). Between the two subdomains, most of the secondary-structure elements are conserved with respect to their position in the three-dimensional structure, although they often differ in length. This is particularly obvious for α-helix 1, which comprises just four residues in the N-terminal subdomain but eleven in the M subdomain. Similarly, α-helix 2 has 5 vs. 10 amino-acid residues in the two subdomains. In general, the strands of the central β-sheet appear to align better between the two subdomains than do the α-helices.

Each of the SUD_core_ subdomains is related to the macrodomain of the histone macro2A ([18]; PDB code 1ZR3, molecule C; for SUD-N: Z-score 9.8, r.m.s.d. 2.5 Å for 112 out of 184 Cα atoms, 12% sequence identity; for SUD-M: Z-score 8.6, r.m.s.d. 2.8 Å for 115 out of 184 Cα atoms, 19% sequence identity). Called “X-domains”, single macrodomains are also found in alphaviruses, in hepatitis E virus, and in rubella virus, in addition to coronaviruses [27],[28]. The SARS-CoV X-domain (Nsp3b), the domain immediately preceding the SUD in Nsp3, shares no recognizable sequence identity with SUD-N (12%) or SUD-M (7%) (Figure 2C), but its three-dimensional structure [22],[23] (PDB code 2ACF, chain A) can be superimposed onto each of the two SUD subdomains with an r.m.s.d. (based on Cα atoms) of 2.7 Å and 2.3 Å, respectively (Figure 2D). Thus, within Nsp3, SARS-CoV has three macrodomains aligned one after the other.

**Figure 3:**
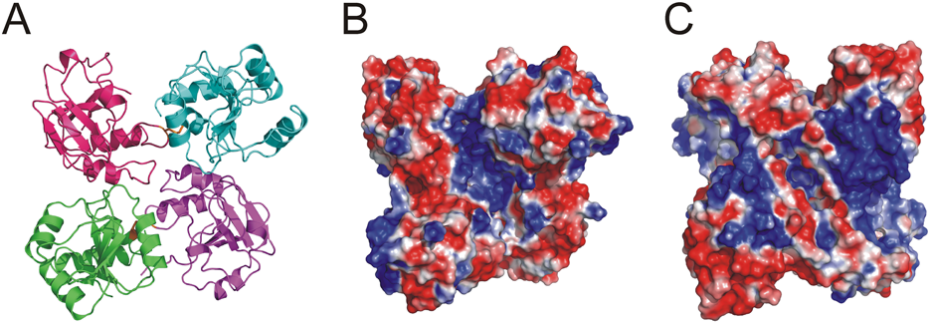
Structure of the SUD_core_ dimer. (A) SUD_core_ forms a head-to-tail dimer. SUD-N and SUD-M of monomer A are colored violet and cyan, respectively, and SUD-N and SUD-M of monomer B are colored magenta and green, respectively. (B) Surface of the SUD_core_ homodimer colored according to electrostatic potential (blue, positive potential; red, negative potential). Orientation is the same as in the cartoon representation in (A). The extended patches of positive potential (blue) are possible binding sites for G-quadruplexes or other nucleic acids. (C) As (B), but rotated by 180°. The narrow cleft running across the dimer surface (with a ∼45° orientation relative to the monomer-monomer interface, which runs horizontal in this illustration) could be a potential protein-binding site. The monomer– monomer interface is largely hydrophilic and buries ∼1130 Å^2^ of exposed surface per monomer.

In both crystal forms, SUD_core_ displays the same head-to-tail dimer, with the SUD-N subdomain of monomer A interacting with the SUD-M subdomain of monomer B, and *vice versa*. Approximately 1130 Å^2^ of solvent-accessible surface per monomer is buried upon dimerization (Figure 3). Due to the two-domain architecture of each monomer, the resulting four lobes give the dimer a quasi-tetrahedral shape (Figure 3A). Involving ∼10 hydrogen bonds and four well-defined salt-bridges (AspB440…ArgA554, ArgB473…GluA619, ArgB554…AspA440, and GluB619…ArgA473), interactions between the monomers are largely hydrophilic. As to be expected, the structures of the monomers are very similar to one another, with r.m.s.d. values (for Cα atoms) of 0.58 Å between monomers A and B of the monoclinic crystal form, and 0.11–0.37 Å between monomers A–D of the triclinic form. The structure of SUD-M alone is even better conserved between the individual copies of SUD_core_. Also, the fold of the SUD-M subdomain is similar to the model of the SUD fragment 527–651 derived from NMR measurements, which was published very recently (r.m.s.d. ∼0.9 Å) [29].

### The SUD_core_ macrodomains fail to bind ADP-ribose

The function of the coronaviral X-domain is still unclear; for some coronaviruses such as HCoV 229E and SARS-CoV, it has been shown to exhibit a low ADP-ribose-1″-phosphate phosphatase (Appr-1″-pase, occasionally also called “ADRP”) activity and to bind the product of the reaction, ADP-ribose [21]–[23],[30]. However, the two subdomains of SUD_core_ do not bind ADP-ribose, as we have demonstrated by zone-interference gel electrophoresis (Figure S1).

### SUD_core_ and its individual subdomains bind G-quadruplexes

**Figure 4:**
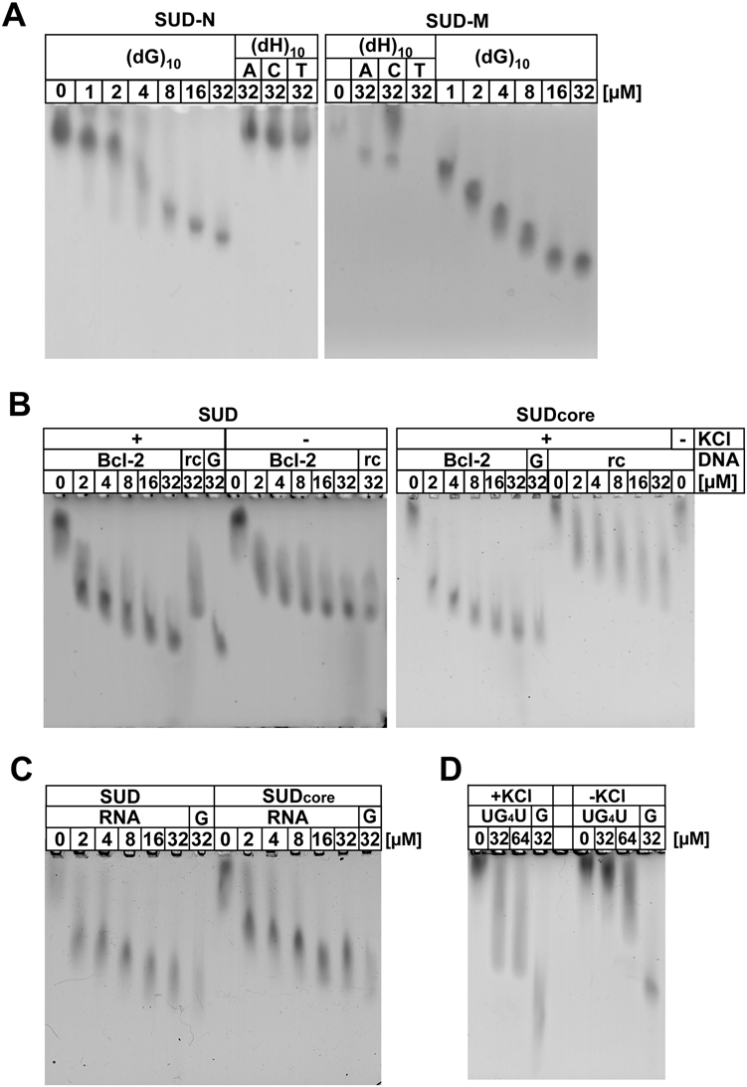
Binding of oligonucleotides to SUD as demonstrated by zone-interference gel electrophoresis. Protein concentration was 10 µM in all experiments. (A) Binding of increasing concentrations (indicated above the lanes) of (dG)_10_ to the SUD-N and SUD-M subdomains of SUD_core_ (left and right panel, resp.). Comparison with 32 µM (dA)_10_, (dC)_10_, or (dT)_10_ shows that the binding is specific for (dG)_10_. “H” stands for A, C, or T. (B) Binding of increasing concentrations (indicated above the lanes) of the quadruplex-forming oligodeoxynucleotide 5′-GGGCGCGGGAGGAATTGGGCGGG-3′ (labeled “Bcl-2”) as occurring within the *bcl-2* promoter region, in the presence and absence of 100 mM KCl, which is known to promote quadruplex formation. Left panel, full-length SUD; right panel, SUD_core_. The reverse-complementary oligodeoxynucleotide (labeled “rc”), which fails to form a quadruplex but exceeds the minimum length of ∼15 nucleotides for non-quadruplex interaction with SUD, is also bound, but with reduced affinity and independently of KCl. (dG)_10_ (labeled “G”) has been included as a positive control. (C) Binding of increasing concentrations (indicated above the lanes) of the quadruplex-forming oligoribonucleotide 5′-UGGGGGGAGGGAGGGAGGGA-3′ (labeled “RNA”) as occurring in the 3′-nontranslated region of chicken elastin mRNA. Left panel: interaction with full-length SUD; right panel: interaction with SUD_core_. Binding of (dG)_10_ (labeled “G”) is shown for comparison. 100 mM KCl was present in all lanes. (D) Binding to SUD_core_ of the quadruplex-forming oligonucleotide 5′-UGGGGU-3′ (labeled “UG_4_U”) in the presence (left) and absence (right) of 100 mM KCl. (dG)_10_ (labeled “G”) has been included as a positive control.

When we investigated possible interactions between SUD and nucleic acids by zone-interference gel electrophoresis, we found that the domain binds oligo(G) and oligo(dG) stretches with a K_D_ of ∼1 µM, but not oligo(dA), (dC), or (dT) [31]. Single-stranded nucleotides of random sequence are only bound if they are longer than ∼15 nucleotides. Here we demonstrate that each of the two individual SUD subdomains also binds oligo(dG) (Figure 4A). With oligo(dH), where H stands for A, C, or T, but not G, only very small gel shifts, if at all, were observed. As oligo(G) stretches are known to form G-quadruplexes, i.e. four-stranded nucleic-acid structures formed by contiguous guanines [32], we also examined the binding to the oligodeoxynucleotide 5′-GGGCGCGGGAGGAATTGGGCGGG-3′, a G-rich sequence present in the *bcl-2* promoter region. This oligonucleotide has been shown by NMR spectroscopy to form a G-quadruplex ([33]; PDB code 2F8U). We found that both full-length SUD and SUD_core_ do indeed bind this oligodeoxynucleotide and that this process is enhanced by the addition of K^+^ ions, which are known to stabilize G-quadruplex structures (Figure 4B). In agreement with the ability of SUD to non-specifically bind to oligonucleotides of >15 bases [31], both SUD and SUD_core_ were found to bind the reverse-complementary sequence, but with low affinity and, more importantly, independent of K^+^ ions.

As there is no evidence for SARS-CoV Nsp3 entering the nucleus and binding to DNA, we examined whether SUD would bind to an RNA known to form a quadruplex structure. Indeed, zone-interference gel shift experiments revealed major shifts for both SUD and SUD_core_ in the presence of the oligoribonucleotide 5′-UGGGGGGAGGGAGGGAGGGA-3′, which is a protein-binding element in the 3′-nontranslated region of chicken elastin mRNA [34] and forms G-quadruplexes [35] (Figure 4C). Furthermore, we observed a significant gel shift for SUD_core_ when we added the short oligonucleotide UGGGGU, which has also been shown to form a G-quadruplex ([36]; PDB code 1J8G). This shift was also enhanced by the addition of K^+^ (Figure 4D). Thus, SUD binds RNA (rG)-quadruplexes and DNA (dG)-quadruplexes with comparable affinity.

### Effect of lysine mutations on G-quadruplex binding

**Figure 5:**
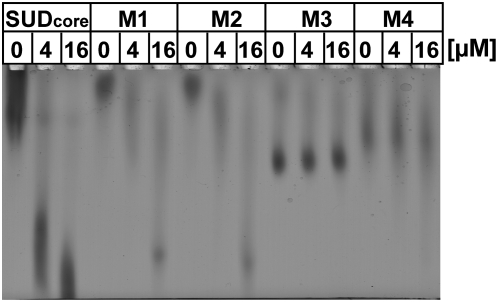
G-quadruplex binding is affected by mutations of lysine residues on the surface of SUD_core_. Binding of double and triple mutants of SUD_core_ to the quadruplex-forming oligodeoxynucleotide 5′-GGGCGCGGGAGGAATTGGGCGGG-3′ as occurring within the *bcl-2* promoter region, in the presence of 100 mM KCl, as demonstrated by zone-interference gel electrophoresis. Protein concentration was 10 µM in all experiments. Oligonucleotide at two concentrations (4 and 16 µM) was added to wild-type SUD_core_ and mutants M1 (K505A+K506A), M2 (K476A+K477A), M3 (K563A+K565A+K568A), and M4 (K565A+K568A+E571A). Mutants M1 and M2 show reduced shifts, in particular at 4 µM nucleotide, whereas mutants M3 and M4 abolish binding. Note that in the absence of nucleotide, mutant proteins M3 and M4 behave differently on the gel because of different charges.

Inspection of the structure of the SUD dimer reveals a central narrow cleft running across the dimer surface, but distinct from the monomer-monomer interface (Figure 3C), which could be a binding site for another protein. In addition, there are several positively charged patches in the center of the dimer (Figure 3B), and on its backside (Figure 3C), which could be involved in binding to G-quadruplexes. We have prepared four sets of mutations by replacing lysine residues (and one glutamate) in these patches by alanines. The first two pairs of mutations, K505A+K506A (M1, at the end of helix αN4) and K476A+K477A (M2, in the loop between αN3 and βN5), are located on the surface of the SUD-N subdomain and lead to reduced shifts with G-quadruplexes in the zone-interference gel electrophoresis experiment, both with the G-quadruplex from the *bcl-2* promoter region (Figure 5) and with (dG)_10_ (not shown). The second set of mutations, K563A+K565A+K568A (M3) and K565A+K568A+E571A (M4) are located in the loop connecting αM2 and βM3 of the SUD-M subdomain and abolish G-quadruplex binding altogether (Figure 5), again with both oligonucleotides.

## Discussion

When the SARS-unique domain was first predicted [1], the boundaries of the domain were set approximately at Nsp3 residues 352 and 726. We made major efforts to produce this protein in a stable form, but with little success. Only when we used *in-vitro* protein synthesis, were we able to obtain small amounts of a relatively stable preparation comprising Nsp3 residues 349–726 [31]. At the N-terminus of this construct, up to eleven residues actually correspond to the C-terminus of the preceding X-domain (Nsp3b). When we expressed a gene construct coding for SUD (349–726) in *E. coli*, we observed rapid proteolytic degradation of the N-terminal segment. The relatively stable intermediate obtained had its N-terminus at Nsp3 residue 389. The N-terminal segment ∼359–388 is predicted to be intrinsically unfolded by several prediction programs (not shown). Therefore, we assume segment 359–388 to be merely a linker between Nsp3b and SUD, and 389 to be the first residue of the latter. This assignment is justified by the observation that in our crystal structures reported here, the SUD-N subdomain is a complete macrodomain without any residues lacking at the N-terminus. Therefore, the protein corresponding to Nsp3 residues 389–726 is called “full-length SUD” here.

In this communication, we describe the crystal structures at 2.2 Å and 2.8 Å resolution (monoclinic and triclinic form, respectively) of the core of the SARS-unique domain (SUD_core_, Nsp3 residues 389–652). SUD_core_ turns out to consist of two subdomains, SUD-N (Nsp3 residues 389–517) and SUD-M (525–652), each exhibiting the fold of a macrodomain. The two subdomains are connected by a flexible linker (residues 518–524) and a disulfide bond. Even though coronavirus replication occurs in the cytosol, where the environment is reductive, it is unlikely that the formation of this disulfide is an artifact owing to handling of the protein: As the linker between the SUD-N and SUD-M subdomains is very short (seven residues), and the mutual orientation of the subdomains is fixed due to the tight dimerization, cysteine residues no. 492 and 623 will be very close to one another irrespective of the exact conformation of the linker. In fact, disulfide bonds are not uncommon in coronaviral non-structural proteins (Nsps) involved in RNA replication or transcription. Among others, they have been observed in HCoV-229E Nsp9 [25] and turkey coronavirus Nsp15 [26], but in these cases, the disulfide bond connects two subunits of the homo-oligomeric proteins, whereas the occurrence in SUD_core_ is the first case of an *intra*molecular disulfide bond described in a coronavirus Nsp.

Coronavirus replication in the perinuclear region of the cell is localized to double-membrane vesicles that have been hijacked from the endoplasmic reticulum or late endosomes [37]–[40]. These vesicles are around 200–350 nm in diameter and present alone or as clusters in the cytosol [38]. The milieu inside or at the surface of these vesicles is unknown, but it is well possible that it is partially oxidative. It has also been speculated [25] that formation of disulfide bonds may be a way for the coronaviral Nsps to function in the presence of the oxidative stress that is the consequence of the viral infection [41]–[43].

Our identification of two macrodomains in SUD_core_ brings the number of these domains in SARS-CoV Nsp3 to three. What are the functions of these modules? The original SARS-CoV “X-domain” (Nsp3b) has been shown to have low ADP-ribose-1″-phosphate phosphatase (Appr-1″-pase or “ADRP”) activity [21]–[23]. However, this assignment is controversial. A nuclear Appr-1″-pase (Poa1p in yeast, [20]) is an enzyme of a tRNA metabolic pathway, but there is no evidence for coronavirus Nsp3 ever being translocated to the nucleus, and the other enzymes involved in this pathway are missing in coronaviruses (with the exception of the cyclic 1″,2″-phosphodiesterase (CPDase) in group 2a viruses such as Mouse Hepatitis Virus, Bovine Coronavirus, and Human Coronavirus OC43). Therefore, it has been proposed that the X-domain may be involved in binding poly(ADP-ribose), a metabolic product of NAD^+^ synthesized by the enzyme poly(ADP-ribose) polymerase (PARP; [23]). However, we have recently demonstrated that the X-domain of Infectious Bronchitis Virus (IBV) strain Beaudette, a group-3 coronavirus, does not have significant affinity to ADP-ribose [24]. This can be explained on the basis of crystal structures: In the X-domain (Nsp3b) of SARS-CoV [23], and in that of HCoV 229E [24], a stretch of three conserved glycine residues is involved in binding the pyrophosphate unit of ADP-ribose, whereas in the corresponding domain of IBV strain Beaudette (but not in all IBV strains, see [44]), the second glycine is replaced by serine, leading to steric interference with ADP-ribose binding [24]. In the two SUD subdomains, the triple-glycine sequence is not conserved (see Figure 2C), and hence, they do not bind ADP-ribose either.

Neuman *et al.* [2] reported that full-length SUD binds cobalt ions, whereas a domain called SUD-C by these authors, which is however almost identical (residues 513–651) to our SUD-M (525–652), does not. From this, they concluded that the metal-binding activity is associated with the cysteine residues in the N-terminal subdomain. We were also able to observe binding of cobalt ions to SUD_core_ by following the occurrence of a peak at 310 nm in the UV spectrum, which, in contrast to the data presented by Neuman *et al*. [2], could be reverted by addition of zinc ions. However, when we removed the N-terminal His-tag, this phenomenon could no longer be observed. Furthermore, we note that of the four cysteine residues in the SUD-N subdomain (residues 393, 456, 492, and 507), 456 and 507 are non-accessible in the interior of the subdomain, and 492 is involved in the buried disulfide bond to Cys623; therefore, Cys393 and perhaps the solvent-exposed His423 would remain the only potential ligands for cobalt ions in SUD-N. However, these residues are >12 Å apart and thus unlikely to chelate cobalt ions.

For SUD-M, a recent publication [29] reported binding to oligo(A). However, we fail to observe this (Figure 4A, lane labeled “A”). Instead, we have demonstrated that full-length SUD and SUD_core_ bind oligodeoxynucleotides and oligoribonucleotides that form G-quadruplexes. For full-length SUD and SUD_core_, we had previously shown binding to oligo(dG) and oligo(G) stretches [31], but the demonstration here of oligo(dG) binding to the individual SUD_core_ subdomains, SUD-N and SUD-M, is unexpected because their overall electrostatic properties are very different from one another: SUD-N is acidic (pI = 5.3), whereas SUD-M is basic (pI = 9.0). However, even SUD-N shows surface patches with positive electrostatics that could bind nucleic acid (Figure 3B).

We have used automatic docking procedures to place the G-quadruplex found in the *bcl-2* promoter region ([33]; PDB code 2F8U) into our crystal structures. One potential binding site identified is in the cleft between the SUD-M and the SUD-N subdomains within the SUD_core_ dimer (Figure S2A); this binding site is spatially close to the mutations M3 and M4, consistent with the observation that these mutations abolish binding completely. However, we have previously shown by Dynamic Light-Scattering that G-quadruplex binding leads to oligomerization of SUD_core_ [31]. Consequently, we have also constructed models based on the packing modes of SUD_core_ dimers observed in our crystal structures. One potential binding site for G-quadruplexes might be in a cleft between two consecutive SUD_core_ dimers as they occur in both the monoclinic and triclinic crystal forms (Figure S2B), but for confirmation, any of these models will have to await crystallographic determination of the complex. In summary, our mutation experiments demonstrate an involvement of several of the many lysine residues of SUD in binding G-quadruplexes, but as it is probably extended surfaces of SUD_core_ oligomers that participate in this process, it is not possible to pinpoint any single amino-acid residue.

The target of SUD binding could be G-quadruplexes in RNA of viral or/and cellular origin. The SARS-CoV genome contains three G_6_-stretches (one on the plus-strand and two on the minus-strand) and an additional two G_5_-sequences, which could perhaps form local G-quadruplexes. However, the G-stretch binding capabilities of SUD and SUD_core_ seem to have been optimized for recognition of longer G-rich sequences. By systematic variation of the length of oligo(dG), we found that SUD_core_ exhibits strongest affinity (K_D_ ∼0.45 µM) for (dG)_10_ to (dG)_14_ [31]. The 3′-nontranslated regions of several host-cell mRNAs coding for proteins involved in the regulation of apoptosis and in signaling pathways contain long G-stretches and could also be targets of SUD. Examples of such mRNAs are those coding for the pro-apoptotic protein Bbc3 [45], RAB6B (a member of the Ras oncogene family, [46]), MAP kinase 1 [47], and TAB3, a component of the NF-κB signaling pathway [48]. It is conceivable that these proteins might be targets for the virus when interfering with cellular signaling. Changes in the stability and/or translation efficiency of these mRNAs due to the binding of a viral regulatory factor could result in an altered reaction of the infected cell to apoptotic signals, or it could silence the antiviral response.

The idea that coronaviral X-domains might function as modules binding poly(ADP-ribose) [23] received support from the observation that some macrodomains are connected with domains showing poly(ADP-ribose) polymerase (PARP) activity, i.e. in the so-called macroPARPs (PARP-9 and PARP-14) [49]. There are 18 human genes for members of the PARP family; the prototype enzyme, PARP-1, catalyzes the post-translational modification of many substrate proteins, including itself, in a multitude of cellular processes (DNA repair, transcriptional regulation, energy metabolism, and apoptosis) [50]–[52]. Interestingly, SUD-M and the C-terminal 74-residue subdomain (SUD-C) that is missing in our SUD_core_ construct together show a ∼15% sequence identity (32% similarity) to the catalytic domain of PARP-1. However, the three-dimensional structures of SUD-M (this work) and the C-terminal domain of PARP-1 [53] are different and cannot be superimposed. Another feature common between SARS-CoV SUD and PARP-1 is that the latter has recently been shown to bind to G-quadruplexes [54], although it is generally assumed that this occurs through the DNA-binding domain rather than the catalytic domain of PARP-1.

PARP-1 and most of its family members are located to the nucleus, while PARP-4 and others predominantly act in the cytoplasm [50]–[52]. PARP-4 is incorporated into vaults, RNA-containing subcellular particles in the cytoplasm [55]. Furthermore, ZAP, a human antiviral protein comprising a C-terminal PARP-like domain devoid of catalytic activity, has been shown to exhibit antiviral activity on alphaviruses [56], which contain an X-domain similar to that of coronaviruses [23],[27],[28]. In addition, ZAP contains an N-terminal zinc-finger domain, a central TiPARP (2,3,7,8-tetrachlorodibenzo-*p*-dioxin (TCDD)-inducible PARP) domain, and a WWE domain (a protein-protein interaction module in ubiquitin and ADP-ribose conjugation proteins). In fact, ZAP appears to be part of the human innate immune system and to play a role comparable to APOBEC3G in HIV infection [57]. It is possible that this group of viruses has evolved macrodomains to counteract the antiviral activity of ZAP. Indeed, macrodomains can inhibit PARPs, as has been shown for the macrodomain of the histone mH2A1.1, which downregulates the catalytic activity of PARP-1 [58]. Having three macrodomains at its disposal, SARS-CoV may be much more efficient in knocking down the antiviral response of the host cell than other coronaviruses. Whether this involves a direct interaction between SUD and ZAP or another member of the PARP family, or competition for G-quadruplexes in viral or host-cell RNA, remains to be shown.

## Materials and Methods

### Recombinant protein production and purification

Full-length SUD (Nsp3 residues 389–726) and the fragment SUD_core_ (Nsp3 residues 389–652, previously called “SUDc5b”) of SARS-CoV strain TOR2 (acc. no. AY274119) were produced recombinantly in *E. coli* as described [31]. The coding regions for the SUD-N subdomain (Nsp3 residues 389–524) and the SUD-M subdomain (Nsp3 residues 525–652) were constructed by introducing an appropriate deletion into the previously described plasmid pQE30-Xa-c5b [31] using site-directed mutagenesis. Plasmids encoding SUD-N and SUD-M were prepared using primers listed in Table S1. The coding regions for four sets of mutations of SUD_core_, M1 (K505A+K506A), M2 (K476A+K477A), M3 (K563A+K565A+K568A), and M4 (K565A+K568A+E571A), were constructed by introducing appropriate mutations into plasmid pQE30-Xa-c5b [31] using site-directed mutagenesis. Plasmids encoding these mutants were prepared using primers also listed in Table S1. All plasmids provided an N-terminal His-tag and a short linker sequence encoding a factor-Xa cleavage site. The coding regions of the expression plasmids were verified by DNA sequencing. *E. coli* M15 (pRep4) was used as expression host for these constructs. SUD-N, SUD-M, and the mutated proteins were purified according to the same protocol as for SUD_core_ [31].

### Crystallization

SUD_core_ displayed >95% purity in SDS-PAGE, and monodispersity in Dynamic Light- Scattering. Initial crystallization screening was performed using the sitting-drop vapor-diffusion method in 96-well Intelli-Plates (Dunn Laboratories). Several commercial kits (Sigma, Jena Bioscience) were used for the screening. The protein concentration was 6 mg/ml. Using a Phoenix robotic system (Art Robbins), drops were made of 260 nl protein and 260 nl precipitant solution. The optimized crystallization condition consisted of 20% polyethylene glycol monomethyl ether 5000 and 0.2 M ammonium sulfate in 0.1 M morpholinoethane sulfonic acid (pH 6.5). Plate-like crystals grew in 3–5 days, to maximum dimensions of 0.02×0.02×0.01 mm^3^.

### Data collection and processing

Many SUD_core_ crystals had to be tested for diffraction until one yielding data to 2.2 Å resolution was found. The best diffracting crystals belonged to space group P2_1_. Under the same crystallization conditions, a second crystal form belonging to space group P1 was observed, diffracting to lower resolution of about 2.8 Å. Crystals were cryoprotected in reservoir solution that included 30% glycerol, and were harvested into a loop prior to flash-cooling in liquid nitrogen. All data were collected at 100 K from a single crystal each at beamline BL14.2, BESSY (Berlin, Germany), using an MX225 CCD detector (Rayonics), or at beamline I911-2 at MAX-lab (Lund, Sweden), using a Mar165 CCD detector (Marresearch). Data were processed with MOSFLM [59], and reduced and scaled using the SCALA [60] program from the CCP4 suite [61]. Crystals belonging to space group P2_1_ had unit-cell parameters *a* = 46.36 Å, *b* = 68.55 Å, *c* = 94.21 Å, *β* = 99.17°, those belonging to space group P1 had unit-cell parameters *a* = 68.68 Å, *b* = 75.52 Å, *c* = 80.54 Å, α = 77.16°, *β* = 75.61°, γ = 74.48°. Data-collection statistics for both crystal forms are shown in Table 1. The asymmetric unit of the P2_1_ form contained two SUD_core_ monomers, giving a Matthews coefficient [62] of 2.5 Å^3^ Da^−1^ and a solvent content of 51%, whereas the P1 crystal form had four monomers per asymmetric unit, giving corresponding parameters of 3.2 Å^3^ Da^−1^ and 63%.

### Structure determination

We attempted to solve the structure by molecular replacement into the P2_1_ form using the NMR coordinates of a subdomain comprising SARS-CoV Nsp3 residues 513–651; PDB code 2JWJ [29],[63]), which is almost identical to the SUD-M subdomain of SARS-CoV Nsp3. Using the program Phaser [64],[65], we could find two solutions, and the C-terminal part of SUD_core_ was well defined in the electron-density maps. However, for the N-terminal half, only a few segments of poly(Ala) chain could be built into the maps. This starting model was then refined in BUSTER-TNT [66] using Local Structure Similarity Restraints (LSSR) [67] as non-crystallographic symmetry (NCS) restraints to give R and R_free_ values of 0.453 and 0.479, respectively. The resulting 2mF_o_-DF_c_ electron density was subjected to density modification using solvent flattening, histogram matching, and 2-fold NCS-averaging using DM [68]. The averaging masks were calculated and updated using the auto-correlation procedure [69] as implemented in DM. Using the automatic building program BUCCANEER [70] together with REFMAC [71] (as implemented in the CCP4i [72] interface for CCP4) in an iterative procedure for 20 cycles resulted in a model for 501 residues in 10 chains (the longest having 208 residues), in which 448 residues were assigned both a chemical identity and a sequential residue number, while the remaining 53 residues were modeled as poly(Ala) in 8 shorter chains. The R and R_free_ values resulting from REFMAC were 0.374 and 0.414, respectively. This model was refined in BUSTER-TNT, again using LSSR as NCS restraints for the common parts in the already sequenced 448 residues of the dimer, to R and R_free_ values of 0.269 and 0.316. The improved electron density was again subjected to density modification using DM as detailed above, but using a lower solvent content of 35% as well as anisotropically scaled observed amplitudes as output by BUSTER-TNT. The resulting density-modified and NCS-averaged map was then used for automatic model building using the iterative BUCCANEER/REFMAC procedure described above. This produced a model with 511 residues in 5 chains with 487 residues sequenced. The R and R_free_ values from REFMAC for this model were 0.289 and 0.326, respectively.

Since the refinements in BUSTER-TNT at that point showed some problematic low correlations between F_o_ and F_c_ at low resolution, the original images collected from the P2_1_ crystal were reprocessed using XDS [73] and SCALA, applying different high-resolution cutoffs for different segments of the collected images. Details for this dataset are given in Table 1. Subsequent refinement of the P2_1_ form with REFMAC, under application of weak NCS restraints, yielded a model with R = 0.211, R_free_ = 0.264. The advanced handling of NCS restraints through LSSR in BUSTER-TNT gave a final model R = 0.211 and R_free_ = 0.268. The final model in the P2_1_ form comprises 513 residues (A389–A516; A524–A652; B393–B519; B526–B652).

Chain A of the P2_1_ form was used for molecular replacement with the program MOLREP [74] into the P1 form. There was an unambiguous solution for four molecules in the asymmetric unit. This model was refined with BUSTER-TNT (using LSSR for NCS restraints) and rebuilt in Coot [75] to final values of R = 0.223 and R_free_ = 0.240. The final model of the P1 form comprises 1014 residues.

The figures were made with PyMOL [76].

### Zone-interference gel electrophoresis (ZIGE)

The zone-interference gel electrophoresis (ZIGE) device was adapted from Abrahams *et al.* [77]. ZIGE assays were performed using a horizontal 1% agarose gel system in TBE buffer (20 mM Tris, 50 mM boric acid, 0.1 mM ethylenediaminetetraacetic acid (EDTA), pH 8.3). The protein was incubated at room temperature for 30 min with different concentrations of oligodeoxynucleotides, such as (dG)_10_ and *bcl-2* promoter region (5′-GGGCGCGGGAGGAATTGGGCGGG-3′), or oligoribonucleotides (5′-UGGGGGGAGGGAGGGAGGGA-3′ and 5′-UGGGGU-3′). The samples were mixed with dimethylsulfoxide (DMSO; final concentration 10% (v/v)) and a trace of bromophenolblue (BPB). These protein-oligonucleotide samples were applied to the small slots. Oligonucleotide with the same concentration as in the small slots was also mixed with DMSO and BPB in 1xTBE buffer and applied to the long slots of the gel (total volume 100 µl). Electrophoresis was performed at 4°C for 1 h with a constant current of 100 mA. Staining was performed as outlined in [77].

### Accession Codes

Protein Data Bank: Coordinates and structure factors have been deposited with accession code 2W2G (P2_1_ crystal form) and 2WCT (P1 crystal form).

## Supporting Information

Figure S1 Zone-interference gel electrophoresis experiment showing that SUD_core_ fails to bind NAD+ and ADP-ribose. SUD_core_ alone (label 0) and decreasing concentrations (1, 0.5, 0.1, 0.05 and 0.02 mM) of NAD^+^, or decreasing concentrations (1, 0.5, 0.1, 0.05 and 0.02 mM) of ADP-ribose.
(0.70 MB DOC)

Figure S2 Alternative models of G-quadruplex binding to SUD_core_, obtained by automated docking into the crystal structures. The SUD-N and SUD-M subdomains are in violet and cyan, respectively, the G-quadruplex as found in the *bcl-2* promoter region (PDB code: 2F8U) is in orange. The pairs of mutations in SUD-N are indicated by green (M1, K505A+K506A) and blue (M2, K476A+K477A) spheres. The M3 set of mutations in SUD-M is indicated by olive (K563A) and orange (K565A+K568A) spheres. The M4 set of mutations, also in SUD-M, is indicated by orange (K565A+K568A) and yellow (E571A) spheres. (A) A possible binding site is in a cleft between monomers in the SUD_core_ dimer. The binding site is close to the lysine residues replaced by the M3 and M4 mutations, compatible with the inability of these mutants to bind G-quadruplexes. (B) A second potential binding site is a cleft between two neighboring SUD_core_ dimers as found in both crystal packing arrangements (space groups P2_1_ and P1). This binding mode is compatible with the observation of SUD_core_ oligomerization upon G-quadruplex binding.
(3.46 MB DOC)

Table S1 Primer sequences used for SUD-N, SUD-M, and four sets of mutants.
(0.01 MB PDF)
