# Supplementary material for: The SARS-Unique Domain (SUD) of SARS Coronavirus Contains Two Macrodomains That Bind G-Quadruplexes
Source: PLoS Pathog. 2009 May 15;5(5):e1000428. doi: 10.1371/journal.ppat.1000428 (PMC2674928; doi:10.1371/journal.ppat.1000428)
Supplement: Figure S1 — Zone-interference gel electrophoresis experiment showing that SUDcore fails to bind NAD+ and ADP-ribose. SUDcore alone (label 0) and decreasing concentrations (1, 0.5, 0.1, 0.05 and 0.02 mM) of NAD+, or decreasing concentrations (1, 0.5, 0.1, 0.05 and 0.02 mM) of ADP-ribose. (0.70 MB DOC) [file ppat.1000428.s001.doc]

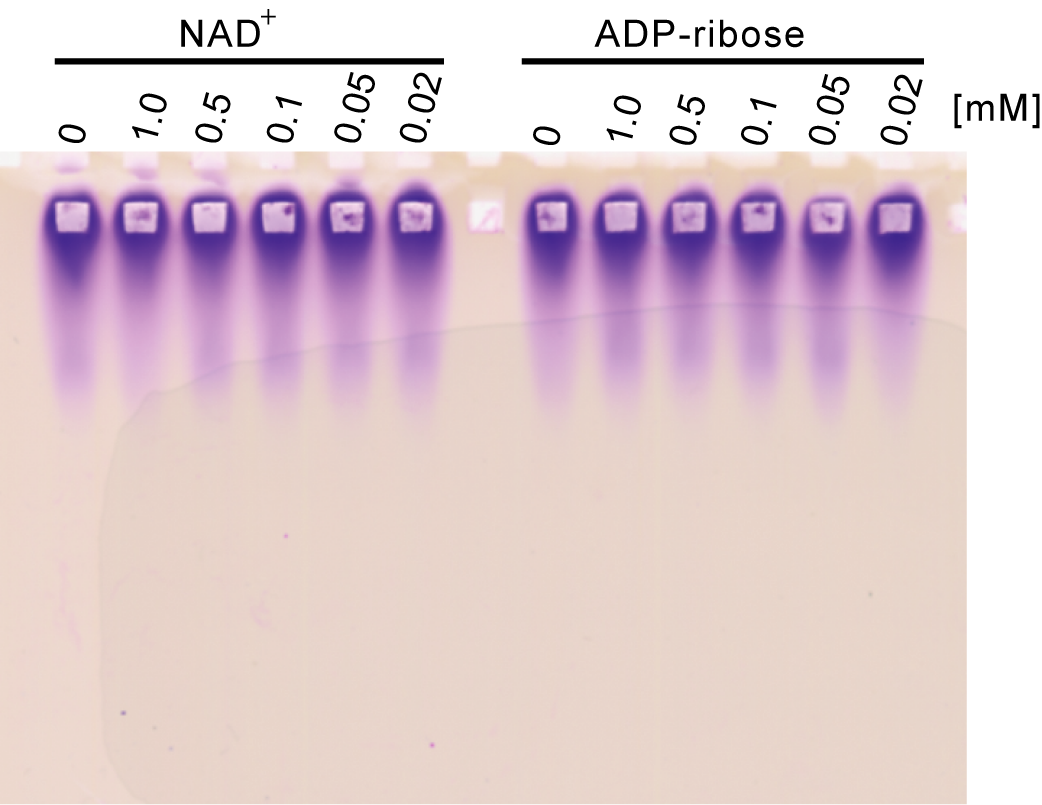


**Figure S1 Zone-interference gel electrophoresis experiment showing that SUD_core_ fails to bind NAD^+^ and ADP-ribose.** SUD_core_ alone (label 0) and decreasing concentrations (1, 0.5, 0.1, 0.05 and 0.02 mM) of NAD^+^, or decreasing concentrations (1, 0.5, 0.1, 0.05 and 0.02 mM) of ADP-ribose.
