# Supplementary material for: The SARS-Unique Domain (SUD) of SARS Coronavirus Contains Two Macrodomains That Bind G-Quadruplexes
Source: PLoS Pathog. 2009 May 15;5(5):e1000428. doi: 10.1371/journal.ppat.1000428 (PMC2674928; doi:10.1371/journal.ppat.1000428)
Supplement: Figure S2 — Alternative models of G-quadruplex binding to SUDcore, obtained by automated docking into the crystal structures. The SUD-N and SUD-M subdomains are in violet and cyan, respectively, the G-quadruplex as found in the bcl-2 promoter region (PDB code: 2F8U) is in orange. The pairs of mutations in SUD-N are indicated by green (M1, K505A+K506A) and blue (M2, K476A+K477A) spheres. The M3 set of mutations in SUD-M is indicated by olive (K563A) and orange (K565A+K568A) spheres. The M4 set of mutations, also in SUD-M, is indicated by orange (K565A+K568A) and yellow (E571A) spheres. (A) A possible binding site is in a cleft between monomers in the SUDcore dimer. The binding site is close to the lysine residues replaced by the M3 and M4 mutations, compatible with the inability of these mutants to bind G-quadruplexes. (B) A second potential binding site is a cleft between two neighboring SUDcore dimers as found in both crystal packing arrangements (space groups P21 and P1). This binding mode is compatible with the observation of SUDcore oligomerization upon G-quadruplex binding. (3.46 MB DOC) [file ppat.1000428.s002.doc]

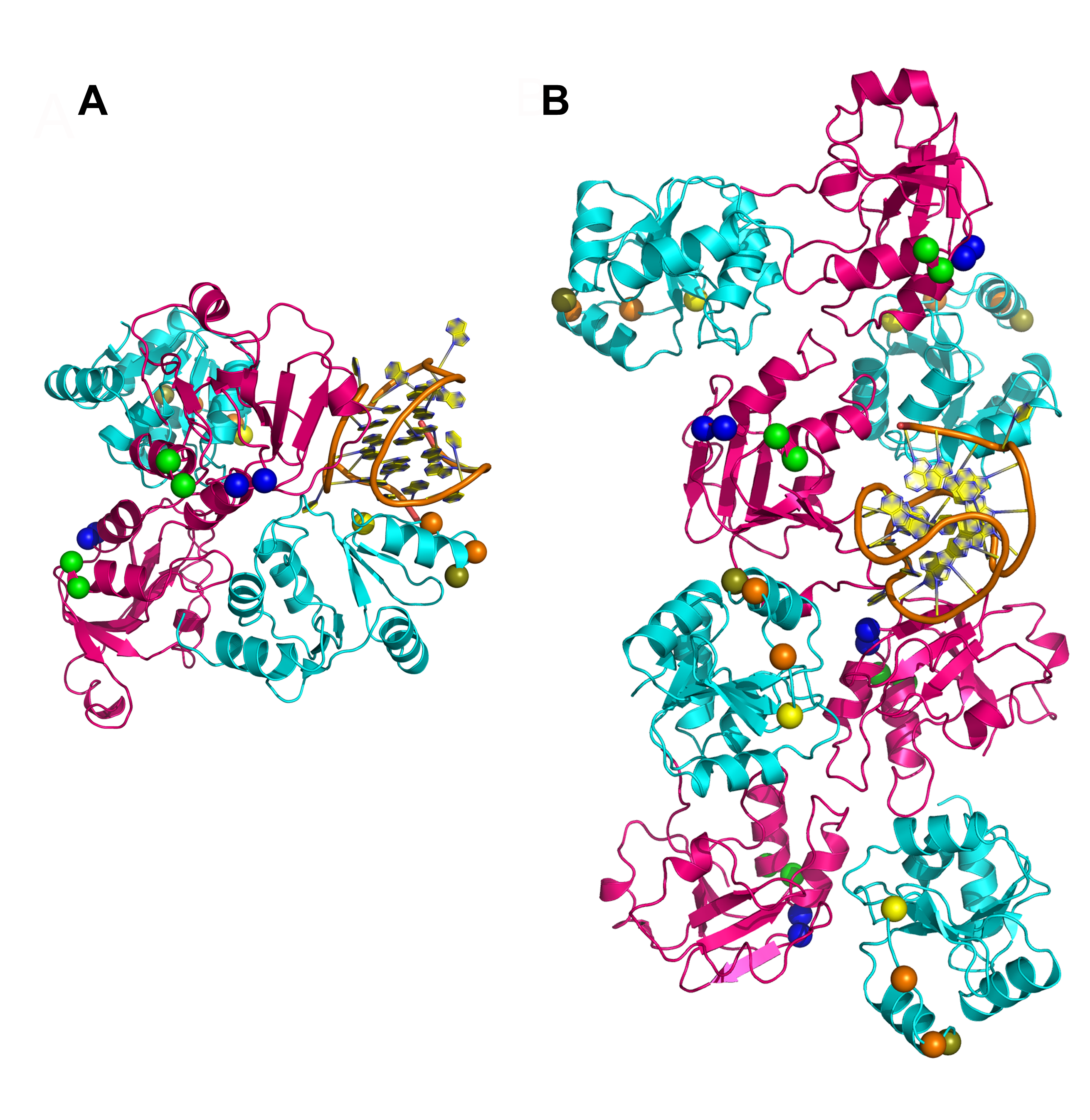


**Figure S2 Alternative models of G-quadruplex binding to SUD_core_, obtained by automated docking into the crystal structures.** The SUD-N and SUD-M subdomains are in violet and cyan, respectively, the G-quadruplex as found in the *bcl-2* promoter region (PDB code: 2F8U) is in orange. The pairs of mutations in SUD-N are indicated by green (M1, K505A+K506A) and blue (M2, K476A+K477A) spheres. The M3 set of mutations in SUD-M is indicated by olive (K563A) and orange (K565A+K568A) spheres. The M4 set of mutations, also in SUD-M, is indicated by orange (K565A+K568A) and yellow (E571A) spheres. **(A)** A possible binding site is in a cleft between monomers in the SUD_core_ dimer. The binding site is close to the lysine residues replaced by the M3 and M4 mutations, compatible with the inability of these mutants to bind G-quadruplexes. **(B)** A second potential binding site is a cleft between two neighboring SUD_core_ dimers as found in both crystal packing arrangements (space groups P2_1_ and P1). This binding mode is compatible with the observation of SUD_core_ oligomerization upon G-quadruplex binding.
