# Supplementary material for: The SARS-Unique Domain (SUD) of SARS Coronavirus Contains Two Macrodomains That Bind G-Quadruplexes
Source: PLoS Pathog. 2009 May 15;5(5):e1000428. doi: 10.1371/journal.ppat.1000428 (PMC2674928; doi:10.1371/journal.ppat.1000428)
Supplement: Table S1 — Primer sequences used for SUD-N, SUD-M, and four sets of mutants. (0.01 MB PDF) [file ppat.1000428.s003.pdf]

Table S1. Primer sequences used for SUD-N, SUD-M, and four sets of mutants.

Name	Sense	Antisense
SUD-N	5'-pTAACCTTGTAATGGAACTGG ATCCGC-3'	5'-pCTCTTCCTTAGCATTAGGTGC TTCTGAAGG-3'
SUD-M	5'-pATTCTAGGAACTGTATCCTG GAATTTGAGAGAAATGCTTGCTC ATGC-3'	5'-pCCTTCCCTCGATACCAGATCC AGAGCCAGATCCGTG-3'
SUD_{core}-M1	5'-pGCTAAGACTGCTCTTGCGGC ATGCAAATCCGCATTTTAT-3'	5'-pATAAAATGCGGATTTGCATG CCGCAAGAGCAGTCTTAGC-3'
SUD_{core}-M2	5'-pCTCTCAAGAGCTTTGGCGGC AGTGCCAGTTGATG-3'	5'-pCATCAACTGGCACTGCCGCC AAAGCTCTTGAGAG-3'
SUD_{core}-M3	5'-GCCATAATGGCAACCATCCAA CGTGCGTATGCAGGAATTGCAAT TCAAGAGGGCATC-3'	5'-GATGCCCTCTTGAATTGCAATT CCTGCATACGCACGTTGGATGGT TGCCATTATGGC-3'
SUD_{core}-M4	5'-CGTAAGTATGCAGGAATTGCA ATTCAAGCGGGCATCGTTGACTA TGGTG-3'	5'-CGATGCCCGCTTGAATTGCAA TTCCTGCATACTTACGTTGGATGG TTGC-3'
